# Improving arrhythmic risk prediction using cardiac magnetic resonance within deep learning in ischemic heart disease

**DOI:** 10.1038/s44325-026-00142-5

**Published:** 2026-07-05

**Authors:** Ahmet Sen, Richard E. Jones, Holly Morgan, Hassan Zaidi, Brian P. Halliday, Daniel J. Hammersley, Amedeo Chiribiri, Divaka Perera, Sanjay K. Prasad, Martin J. Bishop

**Affiliations:** 1 King’s College London, School of Biomedical Engineering & Imaging Sciences, London, UK; 2 Imperial College London, National Heart and Lung Institute, London, UK; 3 Cardiovascular Magnetic Resonance Unit, Royal Brompton and Harefield Hospitals, Guy’s and St Thomas’ NHS Foundation Trust, London, UK; 4 Anglia Ruskin School of Medicine & MTRC, Anglia Ruskin University, Chelmsford, UK; 5 Mid and South Essex, NHS Foundation Trust, London, UK; 6 King’s College London, British Heart Foundation Centre of Research Excellence at the School of Cardiovascular and Metabolic Medicine & Sciences, London, UK; 7 Part of Guy’s and St Thomas’ NHS Foundation Trust, Royal Brompton and Harefield Hospitals, London, UK; 8 NHS Foundation Trust, King’s College Hospital, London, UK

**Keywords:** Biomarkers, Cardiology, Computational biology and bioinformatics, Diseases, Medical research

## Abstract

Sudden arrhythmic death remains a major clinical risk in ischemic heart disease (IHD), underscoring the need for improved risk stratification. Late gadolinium enhancement cardiac magnetic resonance (LGE-CMR) provides measures of scar burden and heterogeneity, but its incremental prognostic value beyond conventional markers such as left ventricular ejection fraction remains uncertain. We analysed two independent IHD cohorts (Dataset 1: *n* = 399, 54 events; National Research Ethics Service approvals 07/H0708/83 and 09/H0504/104+5; Dataset 2: *n* = 424, 50 events; derived from the prospectively registered REVIVED-BCIS2 trial, ISRCTN45979711, registered 20 November 2012)using clinical and LGE-CMR-derived variables to evaluate the contribution of LGE-CMR features, and compare machine learning-based survival modelling approaches. A brute-force feature-selection strategy identified optimal predictor subsets for Cox proportional hazards models, Random Survival Forests, and DeepSurv, evaluated using cross-cohort and pooled validation strategies. Scar entropy consistently emerged as a strong predictor of major arrhythmic events. Non-linear approaches outperformed Cox regression, with DeepSurv demonstrating superior generalization across cohorts and Random Survival Forests showing robust performance in pooled analyses. These findings support scar heterogeneity as an important prognostic marker and suggest that machine-learning survival models may improve arrhythmic risk prediction in patients with IHD.

## Introduction

Sudden arrhythmic events are common and life-threatening occurrences in patients with cardiovascular disease, contributing substantially to overall cardiac mortality worldwide^1–3^. Although they occur less often than non-arrhythmic cardiovascular events, such as heart failure or ischemic episodes, arrhythmic events are unpredictable and carry a high risk of sudden death, emphasizing the need for more accurate risk stratification^4,5^. Patients with impaired left ventricular (LV) function and myocardial scar appear to be at the highest risk of arrhythmic events; however, accurate risk prediction remains challenging. This difficulty stems in part from the limited discriminatory power of conventional clinical features, which often fail to fully capture arrhythmic vulnerability. To address this gap, imaging-derived markers of myocardial scar—such as peri-infarct zone (PIZ) volume and measures of scar heterogeneity (e.g., entropy)^6,7^—have been shown to enhance the precision of arrhythmic risk prediction^8^. Despite advances such as these, the cornerstone determinant of arrhythmic risk and Implantable Cardioverter Defibrillator (ICD) candidacy in clinical practice remains the identification of impaired left ventricular ejection fraction. This approach has recognized limitations, most importantly including underestimating risk in patients with intermediate or normal Left Ventricular Ejection Fraction (LVEF) but high-risk scar features^5,9^.

Early attempts at arrhythmic risk stratification typically relied on simple statistical approaches such as single-variable thresholds, univariate risk markers or group-based comparisons using Kaplan-Meier curves^10,11^. While these methods provided important initial insights, they were limited by their inability to account for multiple interacting predictors or the time-dependent nature of arrhythmic events^12^. As the complexity of cardiovascular risk became more evident, a growing need emerged for approaches capable of incorporating censored data and modelling event occurrence over time. In this context, survival analysis has emerged as the predominant framework for risk stratification, offering a principled means of characterizing time-to-event outcomes and enabling more refined assessments of arrhythmic risk^13,14^.

Survival analysis provides a robust framework for modelling time-to-event outcomes while accounting for censored observations. The Cox proportional hazards model (CoxPH) remains the most widely used approach due to its semi-parametric formulation; however, it relies on assumptions such as proportional hazards and linear covariate effects, which may not fully capture the complex and heterogeneous mechanisms underlying arrhythmic events^15–19^.

To address these limitations, machine learning-based survival models have been developed to capture nonlinear relationships and interactions among predictors^17,20–22^. Random Survival Forests (RSF) provide a flexible^23^, tree-based approach that does not rely on proportional hazards assumptions, while DeepSurv extends Cox modelling using neural networks to learn nonlinear risk functions^23,24^. These approaches enable more flexible modelling of complex clinical and imaging-derived predictors, supporting improved risk stratification in heterogeneous populations.

In this study, we sought to evaluate the utility of advanced survival models for predicting arrhythmic events in patients with ischemic heart disease. We analyzed two independent prospective cohorts of patients with ischemic heart disease, including clinical characteristics and LGE-CMR features of myocardial structure, function and scar. These complementary datasets differ in patient characteristics, imaging vendors, and clinical management, enabling assessment of model robustness and generalizability across heterogeneous clinical settings. Using these datasets, we systematically compared the performance of the CoxPH model, RSF and DeepSurv in predicting arrhythmic events. Beyond overall predictive performance, we also investigated the relative contribution of individual features to risk estimation, aiming to identify which clinical and imaging variables provide the greatest prognostic value. Through this comparative framework, our goal was to advance understanding of how feature-based survival modelling can refine arrhythmic risk stratification and inform future strategies for individualized prevention.

## Results

### Feature selection

**Fig. 1:**
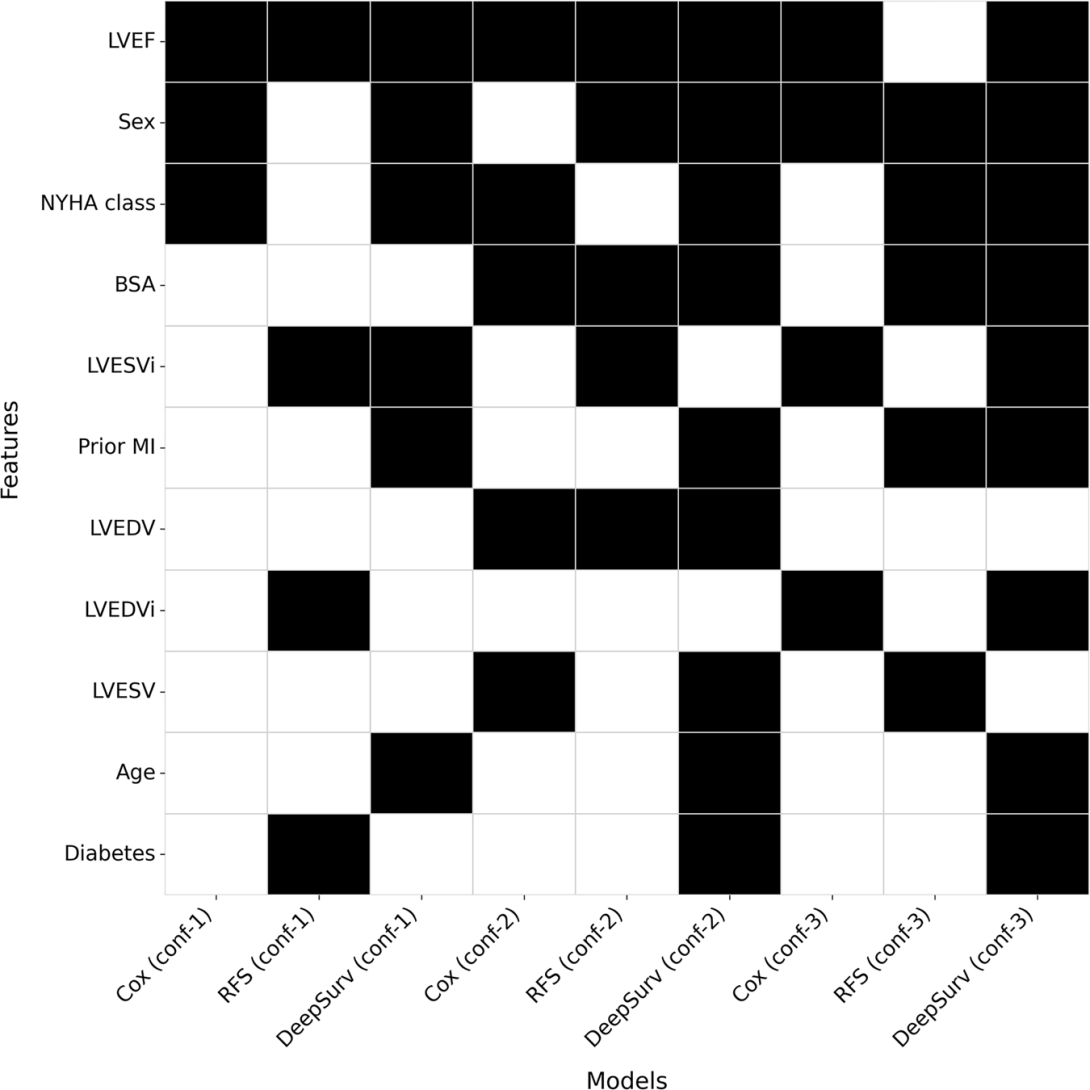
Feature inclusion across survival models and dataset configurations using clinical variables. The heatmap shows the selection of individual features for CoxPH, RSF, and DeepSurv models under three train--test configurations. Rows correspond to candidate predictors, and columns represent model--configuration combinations. Dark cells indicate selected features, whereas light cells indicate features that were not selected.

**Fig. 2:**
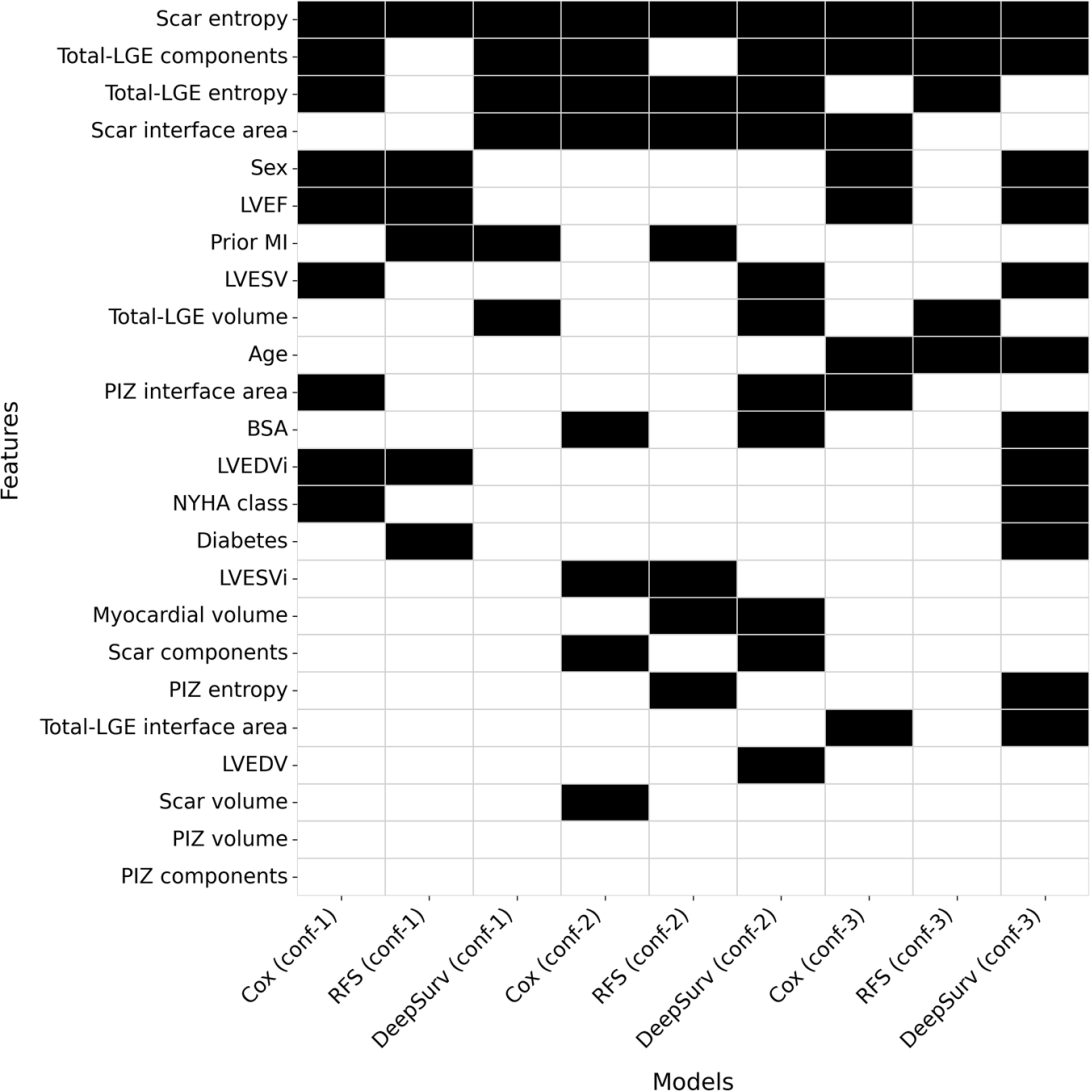
Feature inclusion across survival models and dataset configurations using clinical and imaging variables. The heatmap shows the selection of individual features for CoxPH, RSF, and DeepSurv models under three train--test configurations. Rows correspond to candidate predictors, and columns represent model--configuration combinations. Dark cells indicate selected features, whereas light cells indicate features that were not selected.

The heatmaps (Figs. 1 and 2) summarize the features selected in the best-performing subsets for each model-configuration pair, separately for (i) clinical variables only and (ii) combined clinical and imaging-derived variables. This comparison highlights how feature selection patterns differ between input settings and illustrates the contribution of imaging features to model performance.

The heatmap in Fig. 1 summarizes the selection frequency of clinical variables across models (CoxPH, RSF, and DeepSurv) and configurations (Conf-1 to Conf-3). Overall, several variables were consistently selected across models, indicating their robust contribution to survival prediction. In particular, LVEF, age, and prior myocardial infarction (MI) were among the most frequently selected features, especially in DeepSurv models across all configurations, suggesting their strong and stable prognostic value.

In contrast, variables such as BSA and LVEDV showed more variable selection patterns, being included in some configurations but not consistently across models. RSF exhibited greater variability in feature selection, particularly in Conf-1, where fewer variables were consistently retained compared to CoxPH and DeepSurv. This variability may reflect differences in how each model captures feature interactions and non-linear relationships.

Across configurations, an increasing number of consistently selected variables can be observed from Conf-1 to Conf-3, indicating improved stability of feature selection as more data are incorporated. Notably, DeepSurv demonstrated the most consistent feature selection patterns across configurations, suggesting greater robustness in identifying relevant predictors.

Across all models and configurations, entropy- and intensity-based scar descriptors emerged as the most consistently selected predictors. In particular, scar entropy was the most recurrent feature, appearing in every optimal subset. Other scar heterogeneity descriptors, such as scar interface area, total-LGE interface area, and total-LGE entropy, were also frequently selected, highlighting the prognostic relevance of structural complexity and tissue variability.

Classical volumetric and functional markers retained predictive value but were less consistently chosen. Among these, Left Ventricular End-Diastolic Volume (LVEDV), Left Ventricular End-Systolic Volume (LVESV), and their body surface area-indexed equivalents (LVEDVi, LVESVi) appeared primarily in DeepSurv and CoxPH models, while LVEF was more prominent in all models. Volumetric scar measures (e.g., scar volume, total-LGE volume, myocardial volume) were included in multiple configurations but generally alongside entropy-based features rather than replacing them.

New York Heart Association (NYHA) classes were rarely included across models, particularly in RSF and DeepSurv, suggesting they lack relevance to patient outcomes beyond imaging-derived markers. Demographic variables contributed complementary predictive value, such as age and sex, as well as comorbidities like prior myocardial infarction (MI) and diabetes, which were incorporated in several DeepSurv and CoxPH subsets, suggesting that clinical context augments model performance when integrated with imaging features.

Overall, the heatmap illustrates a clear convergence toward mixed feature subsets, where scar heterogeneity descriptors are combined with functional cardiac indices and clinical covariates. The pattern was consistent across all survival models: RSF typically selected smaller, more parsimonious subsets (4–6 features), while DeepSurv and CoxPH included broader combinations (up to 15). This suggests that non-linear architectures like DeepSurv can exploit richer feature interactions, whereas the ensemble-based RSF attains stability with fewer, targeted predictors. The linear CoxPH model, although less flexible, also favored broader inclusion of covariates.

### Assessment of Survival Models

We comprehensively evaluated the performance of three survival modelling approaches—CoxPH regression, RSF, and DeepSurv—across three distinct dataset configurations (see Section Training and evaluation strategies). Model performance was assessed using complementary metrics capturing both discrimination and calibration, including the C-index, integrated Brier score (IBS), area under the receiver operating characteristic curve (AUC), and time-dependent AUC(*t*) at clinically relevant horizons (2, 5, and 8 years).

Calibration was further evaluated using time-specific calibration plots (at 2, 5 and 8 years) and the integrated calibration index (ICI), which quantifies the mean absolute difference between predicted and observed risks. This multidimensional evaluation framework enabled a balanced assessment of model performance across short-, intermediate-, and long-term prediction horizons.

To investigate the impact of feature composition, models were trained and evaluated under two input settings: (i) clinical variables only, and (ii) a combined set of clinical and imaging-derived variables. This allowed us to assess the incremental value of imaging features in improving predictive performance.

### Clinical variables

In Table 1, *Conf-1*, DeepSurv achieved the highest discrimination (C-index: 0.66 [0.58–0.73]) and lowest prediction error (IBS: 0.071 [0.052–0.090]), outperforming both CoxPH and RSF, which showed similar but lower performance (C-index: 0.61–0.62). Mean AUC followed a similar pattern, with DeepSurv achieving the highest value (0.67 [0.57–0.75]).

**Table 1:** Comparative performance of CoxPH regression, RSF, and DeepSurv across three configurations, reported using C-index, IBS, mean time- dependent area under the ROC curve (AUC) with bootstrap resampling

Conf-1	C-index	IBS	mean AUC
CoxPH	0.61-[0.53–0.69]	0.090-[0.070–0.110]	0.62-[0.53–0.70]
RSF	0.62-[0.54–0.70]	0.094-[0.073–0.115]	0.60-[0.53–0.68]
DeepSurv	0.66-[0.58–0.73]	0.071-[0.052–0.090]	0.67-[0.57–0.75]
**Conf-2**	**C-index**	**IBS**	**mean AUC**
CoxPH	0.69-[0.62–0.76]	0.085-[0.064–0.104]	0.70-[0.63–0.78]
RSF	0.69-[0.62–0.76]	0.087-[0.068–0.106]	0.70-[0.61–0.79]
DeepSurv	0.72-[0.65–0.82]	0.085-[0.067–0.106]	0.73-[0.68–0.80]
**Conf-3**	**C-index**	**IBS**	**mean AUC**
CoxPH	0.73-[0.62–0.83]	0.099-[0.63–0.136]	0.79-[0.69–0.88]
RSF	0.73-[0.62–0.83]	0.091-[0.58–0.125]	0.75-[0.63–0.85]
DeepSurv	0.79-[0.71–0.87]	0.088-[0.60–0.120]	0.85-[0.78–0.91]

In Table 1, *Conf-2*, performance improved across all models. DeepSurv again achieved the highest C-index (0.72 [0.65–0.82]) and mean AUC (0.73 [0.68–0.80]), while CoxPH and RSF showed nearly identical discrimination (C-index: both 0.69). IBS values were comparable across models (0.085–0.087), indicating similar overall prediction error.

In Table 1, *Conf-3*, combining datasets led to further improvements in discrimination. DeepSurv achieved the highest C-index (0.79 [0.71–0.87]) and mean AUC (0.85 [0.78–0.91]), while CoxPH and RSF showed similar performance (C-index: 0.73). IBS values remained comparable across models (0.088-0.099), suggesting broadly similar calibration and overall error.

In Table 2, *Conf-1*, DeepSurv achieved the highest AUC values at 2 and 5 years (0.61 [0.48–0.72] and 0.69 [0.60–0.77], respectively), while RSF showed relatively stronger performance at 8 years (0.64 [0.50–0.70]) compared to CoxPH and DeepSurv. However, variability at longer follow-up was notable, particularly for CoxPH and DeepSurv, as reflected by wider confidence intervals.

**Table 2:** Comparative performance of CoxPH regression, RSF, and DeepSurv across three configurations, reported using area under the ROC curve at time points (AUC(t) 2, 5, and 8 years) with bootstrap resampling

Conf-1	AUC(t) 2y	AUC(t) 5y	AUC(t) 8y
CoxPH	0.56-[0.45–0.66]	0.64-[0.55–0.73]	0.45-[0.30–0.72]
RSF	0.55-[0.45–0.67]	0.64-[0.55–0.72]	0.64-[0.50–0.70]
DeepSurv	0.61-[0.48–0.72]	0.69-[0.60–0.77]	0.62-[0.39–0.84]
**Conf-2**	**AUC(t) 2y**	**AUC(t) 5y**	**AUC(t) 8y**
CoxPH	0.64-[0.50–0.75]	0.70-[0.61–0.79]	0.70-[0.63–0.78]
RSF	0.69-[0.60–0.80]	0.71-[0.62–0.80]	0.68-[0.60–0.76]
DeepSurv	0.71-[0.58–0.82]	0.74-[0.64–0.82]	0.72-[0.65–0.80]
**Conf-3**	**AUC(t) 2y**	**AUC(t) 5y**	**AUC(t) 8y**
CoxPH	0.71-[0.60–0.88]	0.79-[0.68–0.89]	0.80-[0.71–0.89]
RSF	0.67-[0.41–0.75]	0.76-[0.62–0.87]	0.74-[0.59–0.88]
DeepSurv	0.82-[0.74–0.90]	0.83-[0.75–0.90]	0.89-[0.80–0.96]

In Table 2, *Conf-2*, all models demonstrated improved and more stable temporal discrimination. DeepSurv consistently achieved the highest AUC values across all time horizons (0.71–0.74), followed by RSF and CoxPH, which showed comparable performance. Differences between models were modest, with overlapping confidence intervals.

In Table 2, *Conf-3*, overall performance further improved across all time points. DeepSurv exhibited the strongest temporal discrimination, with AUC values increasing over time and reaching 0.89 [0.80–0.96] at 8 years. CoxPH and RSF also showed improved performance compared to earlier configurations, although both remained slightly below DeepSurv, particularly at longer follow-up.

**Fig. 3:**
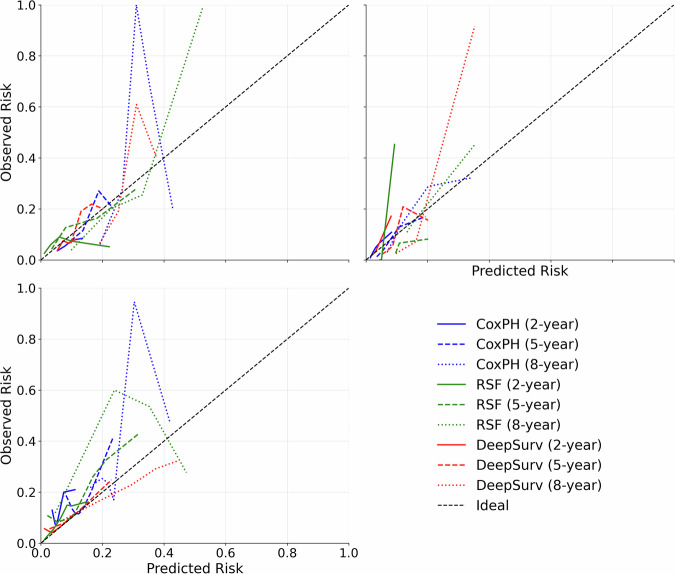
Calibration of survival predictions using clinical variables. Calibration curves are shown for CoxPH, RSF, and DeepSurv across three dataset configurations (Conf-1, top left; Conf-2, top right; and Conf-3, bottom left). Solid, dashed, and dotted lines represent 2-, 5-, and 8-year predictions, respectively. The diagonal dashed line denotes perfect calibration.

Calibration analysis using time-specific calibration curves demonstrated between predicted and observed risks at short- and intermediate-term horizons across all configurations (see Fig. 3 and see Table 3). In Conf-1, CoxPH and DeepSurv showed relatively good calibration at 2 and 5 years (ICI ≈ 0.03–0.05), whereas RSF exhibited slightly larger deviations. At 8 years, calibration became markedly unstable for all models, particularly for CoxPH (ICI = 0.247), reflecting substantial variability in observed risk estimates.

**Table 3:** Calibration performance is further quantified using the integrated calibration index (ICI), reported in the legend for each model and time horizon

	Conf-1			Conf-2			Conf-3		
	ICI 2y	ICI 5y	ICI 8y	ICI 2y	ICI 5y	ICI 8y	ICI 2y	ICI 5y	ICI 8y
CoxPH	0.029	0.028	0.247	0.016	0.024	0.034	0.058	0.047	0.136
RSF	0.056	0.038	0.104	0.031	0.047	0.037	0.043	0.094	0.128
Deepsurv	0.017	0.045	0.107	0.014	0.039	0.046	0.037	0.048	0.100

In Conf-2, calibration improved across all models, with consistently low ICI values at 2 and 5 years (ICI ≈ 0.014–0.047), indicating good agreement between predicted and observed risks. Performance at 8 years remained acceptable, with only moderate increases in ICI (0.034–0.046), suggesting more stable long-term calibration compared to Conf-1.

In Conf-3, calibration remained reasonable at 2 and 5 years across all models, with DeepSurv showing the lowest calibration error at 2 years (ICI = 0.037) and CoxPH at 5 years (ICI = 0.047). However, as in Conf-1, calibration at 8 years showed increased variability (ICI up to 0.136), particularly for CoxPH and RSF.

**Fig. 4:**
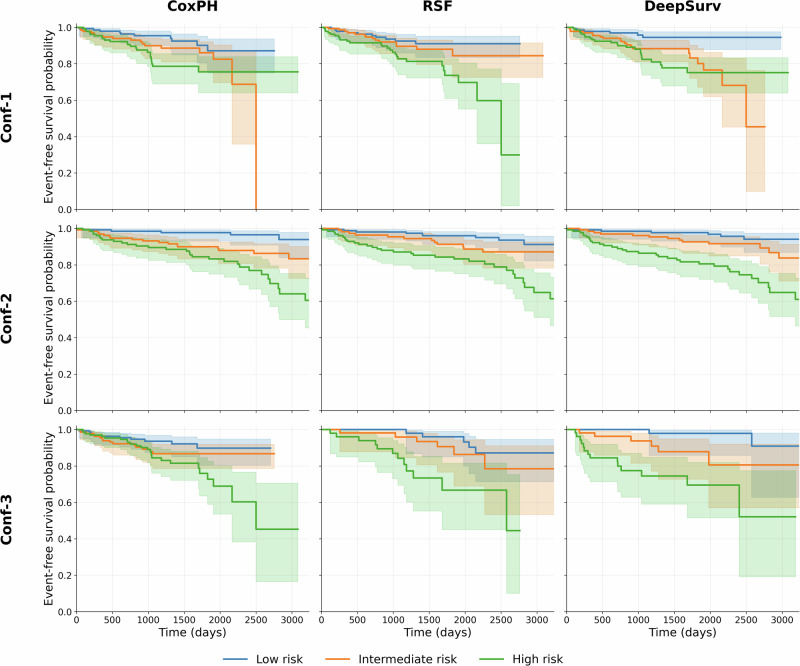
Risk stratification using model-predicted survival probabilities using clinical variables. Kaplan--Meier survival curves stratified patients into low-, intermediate-, and high-risk groups according to predicted risk scores generated by the Cox proportional hazards (CoxPH), random survival forest (RSF), and DeepSurv models. Results are shown for the three dataset configurations (Conf-1, Conf-2, and Conf-3). Shaded regions represent 95% confidence intervals.

Kaplan-Meier survival analysis demonstrated clear stratification of patients into low-, intermediate-, and high-risk groups across all models and configurations (Fig. 4). In *Conf-1*, all models showed significant separation between risk groups, with log-rank *p* values of 5.21 × 10^−3^, 3.06 × 10^−3^, and 7.03 × 10^−4^ for CoxPH, RSF, and DeepSurv, respectively. DeepSurv exhibited the most pronounced separation, particularly between intermediate- and high-risk groups.

In *Conf-2*, risk stratification improved substantially for all models, with highly significant separation between survival curves (CoxPH: 2.29 × 10^−7^; RSF: 5.70 × 10^−6^; DeepSurv: 2.39 × 10^−7^). All three approaches demonstrated clear discrimination across the full risk spectrum, with DeepSurv and RSF showing slightly better separation of intermediate-risk patients compared to CoxPH.

In *Conf-3*, all models maintained significant risk stratification, although separation was slightly reduced for CoxPH (*p* = 4.68 × 10^−2^) compared to RSF (1.43 × 10^−4^) and DeepSurv (3.84 × 10^−5^). Notably, DeepSurv consistently provided the clearest separation between risk groups across all configurations, particularly at longer follow-up times, while RSF also demonstrated robust performance. CoxPH showed comparatively weaker separation, especially in the intermediate-risk group.

Overall, these results indicate that while all models achieved reasonable predictive performance, DeepSurv consistently provided superior discrimination across configurations and time horizons, particularly when trained on larger and more diverse datasets.

### Clinical and imaging variables

In Table 4, *Conf-1*, DeepSurv achieved the highest C-index (0.71 [0.64–0.78]) and lowest prediction error (IBS: 0.070 [0.051–0.088]), outperforming both CoxPH (C-index: 0.65) and RSF (C-index: 0.66). A similar trend was observed for mean AUC, where DeepSurv achieved the highest value (0.73 [0.65–0.80]), indicating improved overall discrimination when imaging features were included.

**Table 4:** Comparative performance of CoxPH regression, RSF, and DeepSurv across three configurations, reported using C-index, IBS, mean time- dependent area under the ROC curve (AUC) with bootstrap resampling

Conf-1	C-index	IBS	mean AUC
CoxPH	0.65-[0.56–0.76]	0.089-[0.069–0.110]	0.67-[0.58–0.75]
RSF	0.66-[0.56–0.76]	0.093-[0.073–0.113]	0.62-[0.55–0.70]
DeepSurv	0.71-[0.64–0.78]	0.070-[0.051–0.088]	0.73-[0.65–0.80]
**Conf-2**	**C-index**	**IBS**	**mean AUC**
oxPH	0.71-[0.64–0.77]	0.096-[0.077–0.114]	0.72-[0.64–0.79]
RSF	0.75-[0.69–0.80]	0.092-[0.075–0.113]	0.75-[0.69–0.91]
DeepSurv	0.76-[0.69–0.82]	0.085-[0.068–0.102]	0.77-[0.70–0.83]
**Conf-3**	**C-index**	**IBS**	**mean AUC**
CoxPH	0.79-[0.71–0.87]	0.095-[0.061–0.134]	0.83-[0.75–0.89]
RSF	0.78-[0.68–0.87]	0.085-[0.055–0.115]	0.80-[0.71–0.88]
DeepSurv	0.84-[0.75–0.92]	0.078-[0.049–0.101]	0.88-[0.80–0.95]

In Table 4, *Conf-2*, performance further improved across all models. DeepSurv again achieved the highest discrimination (C-index: 0.76 [0.69–0.82]; mean AUC: 0.77 [0.70–0.83]), closely followed by RSF (C-index: 0.75). IBS values were lowest for DeepSurv (0.085), suggesting improved calibration and overall predictive accuracy compared to CoxPH and RSF.

In Table 4, *Conf-3*, which combined both datasets, all models demonstrated their highest performance. DeepSurv achieved the strongest results across all metrics (C-index: 0.84 [0.75–0.92]; mean AUC: 0.88 [0.80–0.95]), while CoxPH and RSF showed slightly lower but comparable discrimination (C-index: 0.79 and 0.78, respectively). IBS values were lowest for DeepSurv (0.078), indicating superior overall model performance.

In Table 5, *Configuration 1*, DeepSurv achieved the highest overall discriminative performance across all time horizons. In particular, DeepSurv showed superior short- and intermediate-term discrimination (AUC(2y) = 0.70 [0.57–0.82], AUC(5y) = 0.74 [0.66–0.81]) and maintained the strongest performance at longer follow-up (AUC(8y) = 0.78 [0.69–0.88]). CoxPH and RSF demonstrated comparable performance at 2 and 5 years, while RSF showed reduced long-term discrimination (AUC(8y) = 0.56 [0.48–0.64]).

**Table 5:** Comparative performance of CoxPH regression, RSF, and DeepSurv across three configurations, reported using area under the ROC curve at time points (AUC(t) 2, 5, and 8 years) with bootstrap resampling

Conf-1	AUC(t) 2y	AUC(t) 5y	AUC(t) 8y
CoxPH	0.66-[0.56–0.76]	0.67-[0.58–0.76]	0.63-[0.47–0.87]
RSF	0.65-[0.42–0.82]	0.65-[0.56–0.73]	0.56-[0.48–0.64]
DeepSurv	0.70-[0.57–0.82]	0.74-[0.66–0.81]	0.78-[0.69–0.88]
**Conf-2**	**AUC(t) 2y**	**AUC(t) 5y**	**AUC(t) 8y**
CoxPH	0.67-[0.56–0.76]	0.73-[0.63–0.81]	0.72-[0.64–0.79]
RSF	0.65-[0.52–0.79]	0.77-[0.70–0.83]	0.75-[0.68–0.81]
DeepSurv	0.72-[0.62–0.83]	0.78-[0.70–0.86]	0.77-[0.69–0.84]
**Conf-3**	**AUC(t) 2y**	**AUC(t) 5y**	**AUC(t) 8y**
CoxPH	0.79-[0.71–0.88]	0.84-[0.75–0.90]	0.83-[0.73–0.91]
RSF	0.73-[0.66–0.84]	0.82-[0.71–0.91]	0.79-[0.66–0.91]
DeepSurv	0.87-[0.73–0.95]	0.89-[0.76–0.94]	0.86-[0.75–0.96]

In Table 5, *Configuration 2*, all models showed improved and more consistent performance across time horizons. DeepSurv again achieved the highest AUC values at all time points (AUC(2y) = 0.72 [0.62–0.83], AUC(5y) = 0.78 [0.70–0.86], AUC(8y) = 0.77 [0.69–0.84]). RSF demonstrated strong intermediate- and long-term performance (AUC(5y) = 0.77, AUC(8y) = 0.75), while CoxPH showed slightly lower but stable discrimination across all horizons.

In Table 5, *Configuration 3*, all models achieved their highest performance. DeepSurv substantially outperformed the alternatives, with consistently superior AUC values across all time points (AUC(2y) = 0.87 [0.73–0.95], AUC(5y) = 0.89 [0.76–0.94], AUC(8y) = 0.86 [0.75–0.96]). CoxPH also demonstrated strong performance (AUC(2y) = 0.79, AUC(5y) = 0.84, AUC(8y) = 0.83), slightly outperforming RSF at most time points, although both remained below DeepSurv.

**Fig. 5:**
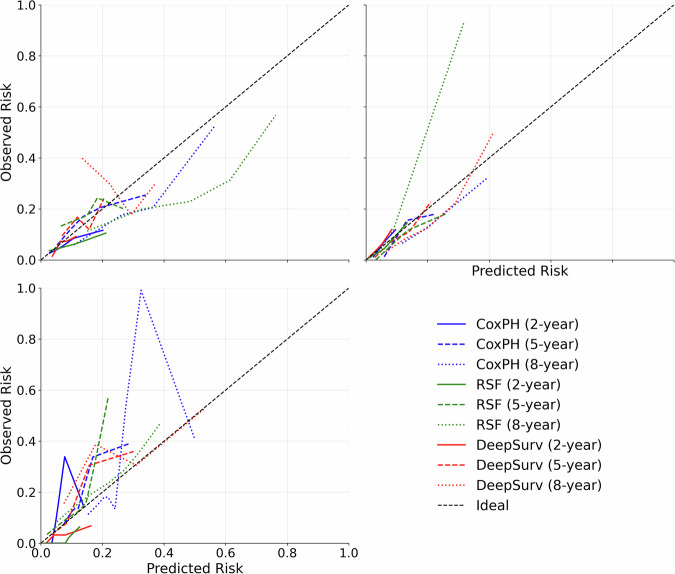
Calibration of survival predictions using clinical and imaging variables. Calibration curves are shown for CoxPH, RSF, and DeepSurv across three dataset configurations (Conf-1, top left; Conf-2, top right; and Conf-3, bottom left). Solid, dashed, and dotted lines represent 2-, 5-, and 8-year predictions, respectively. The diagonal dashed line denotes perfect calibration.

Calibration curves demonstrated agreement between predicted and observed risks at short- and intermediate-term horizons across all configurations (see Fig. 5 and Table 6). In Conf-1, DeepSurv showed the lowest calibration error at 2 years (ICI = 0.013) and remained well calibrated at 5 years (ICI = 0.035), outperforming CoxPH and RSF. CoxPH exhibited slightly higher calibration error at 5 years (ICI = 0.038), while RSF showed larger deviations overall, particularly at 8 years (ICI = 0.186). All models demonstrated increased variability at longer follow-up, with reduced calibration at 8 years.

**Table 6:** Calibration performance is further quantified using the integrated calibration index (ICI), reported in the legend for each model and time horizon

	Conf-1			Conf-2			Conf-3		
	ICI 2y	ICI 5y	ICI 8y	ICI 2y	ICI 5y	ICI 8y	ICI 2y	ICI 5y	ICI 8y
CoxPH	0.029	0.038	0.105	0.010	0.032	0.062	0.063	0.086	0.206
RSF	0.054	0.048	0.186	0.010	0.038	0.064	0.042	0.084	0.182
Deepsurv	0.013	0.035	0.102	0.010	0.038	0.064	0.030	0.014	0.087

In Conf-2, calibration improved across all models and time horizons. Both CoxPH and DeepSurv achieved low calibration error at 2 years (ICI = 0.010), while DeepSurv maintained the best overall calibration across all time points (ICI range: 0.010–0.054). RSF showed slightly higher variability, particularly at 8 years (ICI = 0.064), although overall agreement with observed risk remained acceptable.

In Conf-3, calibration remained strong at 2 and 5 years for all models, with DeepSurv achieving the lowest ICI values at 5 years (ICI = 0.025). CoxPH and RSF showed moderate calibration error at intermediate horizons (ICI up to 0.086), while long-term calibration at 8 years was more variable, particularly for CoxPH (ICI = 0.206) and RSF (ICI = 0.182). DeepSurv demonstrated comparatively better stability at longer follow-up (ICI = 0.087).

**Fig. 6:**
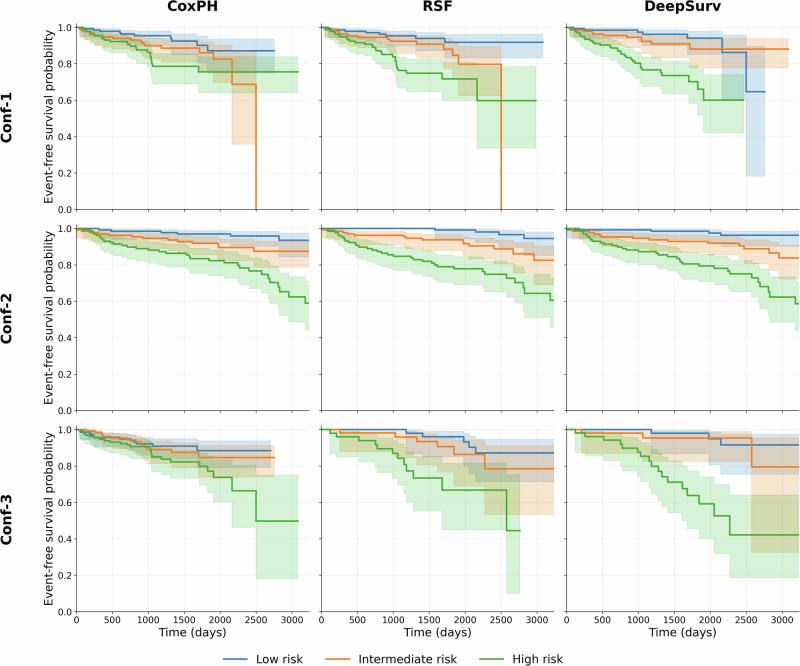
Risk stratification using model-predicted survival probabilities using clinical and imaging variables. Kaplan--Meier survival curves stratified patients into low-, intermediate-, and high-risk groups according to predicted risk scores generated by the Cox proportional hazards (CoxPH), random survival forest (RSF), and DeepSurv models. Results are shown for the three dataset configurations (Conf-1, Conf-2, and Conf-3). Shaded regions represent 95% confidence intervals.

Kaplan-Meier survival analysis demonstrated clear and statistically significant stratification of patients into low-, intermediate-, and high-risk groups across all models and configurations (Fig. 6). In *Conf-1*, all models achieved strong separation between risk groups, with highly significant log-rank *p* values (CoxPH: 5.13 × 10^−4^; RSF: 4.44 × 10^−5^; DeepSurv: 1.46 × 10^−6^). Among these, DeepSurv showed the most pronounced separation, particularly between intermediate- and high-risk groups.

In *Conf-2*, risk stratification further improved, with all models demonstrating very strong discrimination across the full risk spectrum (CoxPH: 1.46 × 10^−7^; RSF: 4.08 × 10^−8^; DeepSurv: 4.10 × 10^−9^). The separation between curves was more consistent than in Conf-1, particularly for DeepSurv and RSF, indicating improved identification of intermediate-risk patients.

In *Conf-3*, all models maintained significant separation between risk groups, with DeepSurv (3.06 × 10^−6^) and RSF (4.71 × 10^−4^) demonstrating stronger discrimination compared to CoxPH (6.72 × 10^−3^). Although separation slightly decreased for CoxPH relative to earlier configurations, DeepSurv continued to provide the clearest distinction between risk groups, especially at longer follow-up durations.

Overall, this comparative evaluation highlights the limitations of CoxPH regression in handling non-linear interactions and complex covariate effects. By contrast, RSF and DeepSurv consistently demonstrated superior or comparable discrimination across multiple time horizons, supporting the adoption of flexible machine-learning approaches for robust and clinically meaningful survival prediction.

## Discussion

In this study, we systematically evaluated the prognostic value of clinical and LGE-CMR features for survival prediction in patients with IHD, comparing three distinct modelling approaches: CoxPH regression, RSF and DeepSurv. By integrating an exhaustive feature selection strategy with rigorous cross- dataset validation, we identified a set of scar predictors—including scar entropy—that consistently contributed to robust risk stratification^25^. Our findings demonstrate that the non-linear model (DeepSurv) often outperformed or matched CoxPH or RSF regression^24,26^ across discrimination and calibration metrics, particularly in capturing the added prognostic value of scar heterogeneity. These results highlight the utility of advanced imaging-derived features and the advantages of flexible, machine learning-based survival models in addressing the complexity of arrhythmic risk prediction.

Several prior studies have investigated survival prediction in structural heart disease using classical statistical models, most commonly Cox proportional hazards regression. For example, recent works have applied Cox-based frameworks to imaging and clinical features for arrhythmic risk stratification^27–30^. While these studies demonstrate the utility of survival modelling, they are constrained by the linear assumptions of Cox regression and limited feature interaction handling. More recently, machine-learning approaches such as RSF have been explored^31^, offering non-linear modelling and improved flexibility. Deep learning-based methods, particularly DeepSurv, have also emerged as promising tools for personalised survival estimation in other areas of cardiovascular medicine^32,33^. However, despite these advances, the utility of deep learning approaches to improve risk prediction in IHD is limited. Many prior studies either rely on a single dataset or, when using an external validation set, report a substantial drop in performance^17^. In contrast, our models achieved consistently strong performance across two independent datasets, an important step toward demonstrating generalizability in survival prediction.

Our work introduces several novel aspects: we benchmarked DeepSurv against both classical and machine-learning alternatives, incorporated a wide range of clinical, volumetric, and microstructural LGE-CMR features, and demonstrated consistent predictive performance across two fully independent datasets. Furthermore, unlike previous studies that typically relied on univariate filtering or penalized regression for feature selection, we employed a brute-force combinatorial search strategy to identify informative subsets of variables, enabling the exploration of potential non-linear feature interactions relevant for survival prediction. While this approach allows a comprehensive evaluation of feature combinations, we did not directly compare it with standard feature selection methods. Therefore, its relative advantage should be interpreted with caution, and future work should include direct comparisons with approaches such as penalized regression to further assess its contribution to predictive performance and robustness.

The concept of a tiered model selection strategy should also be considered in the context of the underlying data structure and research objectives. One of the key motivations for using machine learning-based survival models is their ability to capture non-linear effects and complex interactions between predictors, which may not be adequately represented by traditional regression approaches. In addition, such models can support data-driven hypothesis generation, particularly in settings where prior domain knowledge is limited or where relationships between variables are not well understood.

Accordingly, model selection should not be based solely on predictive performance, but also on the nature of the available data and the intended application. For example, when the goal is exploratory analysis or the identification of novel risk patterns, flexible models such as DeepSurv or RSF may provide important advantages. In contrast, when interpretability, transparency, and clinical implementation are prioritized, simpler models such as CoxPH may be more appropriate. These considerations highlight the importance of aligning model choice with both the complexity of the data and the clinical or research question being addressed.

The feature selection analysis highlights the central prognostic role of scar heterogeneity, particularly entropy and intensity-derived measures. The dominance of scar entropy across all model-configuration pairs suggests that local irregularities in scar texture capture aspects of myocardial remodelling that are not adequately described by simple volumetric indices. Entropy-based descriptors likely reflect microscopic tissue variability, arrhythmogenic substrates, and the spatial distribution of fibrosis, all of which are known contributors to adverse outcomes in structural heart disease^34–36^.

Interestingly, volumetric features such as scar volume and total-LGE volume were included in several high-performing subsets but rarely as standalone predictors. Instead, they tended to co-occur with entropy or intensity features, implying that scar size and heterogeneity provide complementary information: absolute burden reflects disease extent, while heterogeneity reflects substrate vulnerability. This aligns with prior studies^27,37^ suggesting that arrhythmic and survival risk are not solely dependent on infarct size but on its microstructural complexity.

Traditional functional parameters, including LVEF and ventricular volumes (LVEDV, LVESV), were variably selected across models. Their inclusion in CoxPH and DeepSurv indicates that systolic dysfunction remains clinically relevant, but their inconsistent presence compared with entropy-based features suggests that conventional indices may be less discriminative when more detailed tissue characterization is available. Additionally, clinical covariates (such as age, sex, prior MI) were frequently incorporated, reinforcing that clinical status provides an additive prognostic context to imaging-derived features and underscoring the need for a multimodal approach.

Taken together, these findings suggest that scar heterogeneity is a dominant determinant of arrhythmic events, while volumetric, functional, and clinical features provide complementary signals that enhance predictive performance in survival models. The results argue for a shift away from reliance on global functional indices alone, toward integrative models that combine imaging-derived scar complexity with clinical context.

Beyond our findings, the central importance of entropy as a marker of arrhythmogenic substrate is strongly supported by prior work in these same clinical cohorts. Jones et al. demonstrated that scar entropy, along with other microstructural descriptors such as interface area and transmurality, was significantly associated with a primary endpoint of sudden cardiac death in the cohort used here as Dataset 1, highlighting entropy as a key texture-based surrogate of myocardial heterogeneity and conduction complexity^8^. Complementary results were shown by Morgan et al. in their analysis of the cohort used here as Dataset 2, finding that entropy was independently associated with endpoints of all-cause death, cardiovascular death, appropriate ICD therapy, and sustained ventricular arrhythmias—even after accounting for scar volume and conventional risk markers^38^. Notably, their work revealed that entropy was most prognostic in patients with lower total scar, suggesting that microstructural irregularity, rather than scar size alone, may be the principal driver of arrhythmogenic vulnerability in borderline-risk patients. Together, these studies reinforce that entropy captures clinically meaningful aspects of scar organization that are complementary to volumetric measures—strengthening the mechanistic plausibility and translational relevance of our finding that entropy-based heterogeneity dominates feature selection across survival modelling strategies.

These findings are further supported by the direct comparison between models trained on clinical variables alone and those incorporating both clinical and imaging-derived features. Incorporating imaging-derived variables led to consistent improvements in predictive performance compared to clinical variables alone, demonstrating their incremental prognostic value. The addition of imaging information resulted in higher discrimination across all configurations, with notable gains in both C-index and time-dependent AUC, particularly in Conf-3 where larger and more heterogeneous data were available. These improvements were most evident at intermediate and long-term horizons, suggesting that imaging features capture structural and spatial heterogeneity associated with arrhythmogenic substrates that are not fully represented by conventional clinical markers such as LVEF and ventricular volumes.

In contrast, the impact on calibration was more modest. While short- and intermediate-term calibration remained similar between clinical-only and combined models, the inclusion of imaging features contributed to slightly improved stability, particularly for DeepSurv. However, long-term calibration at 8 years remained variable across both input settings, likely reflecting increased censoring and reduced event counts rather than limitations of the feature set.

The comparative analysis revealed a consistent advantage of the neural-network-based survival model across all dataset configurations. DeepSurv demonstrated the strongest overall performance, highlighting its ability to capture non-linear relationships and complex interactions between imaging- and clinical-derived predictors. This superiority was most apparent when richer feature subsets were available, suggesting that the model can effectively leverage higher-dimensional representations to enhance risk prediction. However, this advantage comes with important considerations: DeepSurv requires larger sample sizes, more careful hyperparameter optimization, and greater computational resources. In contexts where sufficiently large datasets are available and interpretability can be balanced against accuracy, DeepSurv represents the most powerful option for long-term outcome prediction.

RSF, while generally less accurate than DeepSurv, demonstrated strong performance with smaller and more parsimonious feature sets. This characteristic makes it particularly attractive when clinical data are incomplete, when imaging-derived features are limited, or when computational simplicity is desired. Moreover, RSF provides natural measures of variable importance, which can help identify key predictors even when the primary goal is not high-precision risk estimation but rather hypothesis generation or exploratory modelling.

CoxPH remains the most interpretable of the three approaches and showed stable long-term discrimination across configurations. Although its predictive performance was lower than RSF and DeepSurv, CoxPH retains clear advantages in clinical translation: it produces easily interpretable hazard ratios, is less prone to overfitting, and requires relatively small datasets to train reliably. For studies emphasizing clinical interpretability and transparency—such as early-phase investigations or guideline-oriented work—CoxPH remains a valuable and pragmatic choice.

Although RSF demonstrated competitive performance and frequently outperformed CoxPH at medium-term prediction horizons, its performance was less stable over longer follow-up periods. This may reflect the increasing impact of censoring and reduced event density, which can affect the stability of tree-based survival estimates in sparsely supported regions of the data. In contrast, CoxPH remained comparatively robust and, in Configuration 3, achieved competitive or superior performance, likely due to its lower variance and greater stability when trained on larger combined datasets. These findings suggest that while flexible models such as DeepSurv and RSF can capture complex non-linear relationships, simpler approaches may retain advantages in stability and long-term risk estimation under certain conditions.

From a clinical perspective, model selection should depend on the intended application. Flexible models such as DeepSurv may be advantageous for individualized patient-level risk prediction when sufficient data and computational resources are available. In contrast, CoxPH may remain preferable in settings where transparency, reproducibility, and ease of implementation are prioritized. RSF may offer a useful balance between flexibility and interpretability. Overall, these findings highlight the importance of considering clinical usability and model stability alongside predictive performance.

An important observation across all models was the progressive improvement from the first to the third configuration, demonstrating the value of refined feature selection in improving prognostic accuracy. Classical regression models benefitted from carefully chosen subsets, but their gains plateaued earlier, whereas DeepSurv continued to improve with richer input information.

Overall, these findings emphasize that modern machine-learning survival models, particularly DeepSurv, provide clear advantages for long-term outcome prediction in complex clinical datasets. Nevertheless, RSF and CoxPH retain important complementary roles: RSF as a strong intermediate-horizon predictor using parsimonious feature sets, and CoxPH as an interpretable, stable baseline that aligns well with established clinical practice.

Taken together, these considerations suggest a tiered model selection strategy. DeepSurv should be the preferred approach when sufficient data and computational resources are available and maximizing predictive accuracy is the priority. RSF offers a strong alternative in resource-limited or data-limited settings, particularly when robust performance with fewer features is required. CoxPH, while less powerful, remains indispensable where interpretability and clinical acceptance are paramount.

This study has several limitations. First, despite combining two independent datasets, the overall sample size remained modest (approximately 400 patients per dataset), with relatively few events (54 in Dataset 1 and 50 in Dataset 2). This limited event count may restrict the power of our models and the stability of feature selection, particularly for more complex architectures. Second, our scar-related features were derived from 2D LGE-CMR slices, whereas myocardial scarring is inherently three-dimensional. Reliance on 2D imaging may therefore introduce inaccuracies in scar quantification and underrepresent complex 3D structures. Third, the resolution of 2D LGE-CMR remains a practical limitation for clinical translation, as voxel size and slice thickness may affect the reliability of entropy and interface measures.

Another important limitation of this study is the variability in baseline scar characteristics between the two datasets. While several scar features were significantly different between patients with and without arrhythmic events in the first dataset, these associations were attenuated or absent in the second dataset. This discrepancy potentially reflects the differences in inclusion criteria, noting that all patients in the REVIVED-BCIS 2 trial had severely reduced LVEF. Although this heterogeneity limits direct comparison of univariate results across datasets, it also highlights the importance of using multivariable modelling and feature selection approaches rather than relying on isolated predictors. By integrating information across multiple features and evaluating performance on independent datasets, our modelling framework mitigates the impact of dataset-specific noise and provides a more robust assessment of prognostic value. Arguably, the high performance of each methodology across both cohorts despite their differences, supports its robustness and potential translatability of the model application across diverse cohorts of patients with IHD.

An interesting observation is the difference in correlation structure among entropy-based features between the two datasets. In Dataset 1, entropy measures were highly correlated, suggesting that they capture similar aspects of scar heterogeneity within a relatively homogeneous ischemic population. In contrast, this pattern was not observed in Dataset 2, where entropy features exhibited lower correlation. This discrepancy may reflect differences in underlying disease phenotype, with Dataset 2 potentially characterized by more diffuse myocardial remodelling and less well-defined scar structure. In such settings, different entropy measures may capture complementary aspects of tissue heterogeneity rather than redundant information.

In addition to biological variability, entropy-based features are sensitive to imaging characteristics, including voxel resolution, noise levels, reconstruction parameters, and segmentation variability. These factors may influence feature stability, particularly in multicentre or multivendor settings. Despite these challenges, entropy features were consistently selected as important predictors across both datasets, providing indirect evidence of their robustness^39–41^. Nevertheless, further work is required to standardize feature extraction and evaluate reproducibility across imaging protocols and centres to support clinical translation.

The extracted scar features differ from conventional radiomics descriptors, as they are designed to capture physiologically meaningful structural characteristics rather than generic texture patterns. However, their reproducibility remains dependent on segmentation quality and imaging characteristics. While a standardized pipeline was applied across datasets, variability in acquisition parameters and segmentation may still influence feature stability, highlighting the need for further validation in multicentre settings.

An important limitation relates to the treatment of non-arrhythmic death as a censoring event. While this approach is consistent with a cause-specific hazard framework, it does not explicitly account for competing risks. In populations with high mortality from non-arrhythmic causes—such as Dataset 2—this may introduce bias, as competing events can preclude the occurrence of arrhythmic outcomes. Future studies should consider competing risk approaches, such as Fine-Gray models^42^, to further refine risk estimation.

A further consideration is the inclusion of correlated predictors, particularly indexed and non-indexed ventricular volume measures. While such variables were retained to allow data-driven feature selection, collinearity may influence the stability of selected features. However, the main findings were robust, as scar heterogeneity features—especially entropy—were consistently selected across models and datasets. In contrast, correlated ventricular volume variables were not co-selected within the optimal feature subsets, suggesting that the feature selection process did not rely on redundant predictors. These observations indicate that the primary conclusions are not driven by collinearity among conventional volumetric variables.

The two datasets included in this study differ substantially in their clinical characteristics, including the prevalence of prior myocardial infarction and distributions of left ventricular function. Dataset 1 is characterized by a higher prevalence of ischemic injury, whereas Dataset 2 appears to represent a population with less focal scar and potentially more diffuse myocardial remodelling. These differences likely influence the relationship between scar-derived features and arrhythmic outcomes. In particular, the attenuation of scar-outcome associations observed in Dataset 2 may reflect a reduced contribution of localized scar heterogeneity in populations where arrhythmogenesis is driven by more diffuse structural or electrophysiological abnormalities. This may also contribute to the reduced predictive performance observed in configurations involving Dataset 2, especially when used as a training set (Configuration 2). These findings highlight the importance of considering cohort-specific disease characteristics when interpreting model performance and generalizability.

Another limitation of the present study is the limited use of advanced explainability techniques for machine learning and deep learning models. Although predictor importance was assessed through feature selection frequency across models and configurations, more detailed interpretability approaches such as SHAP, LIME, or permutation-based analyses were not investigated. These methods may provide additional insight into model behaviour and individual patient-level predictions, particularly for clinically deployable risk stratification frameworks. Future work should therefore explore the integration of explainability techniques to improve interpretability and clinical transparency of ML- and DL-based survival models.

Future work should therefore prioritize validation in larger, more diverse cohorts, ideally integrating multi-center data to capture broader heterogeneity. Expansion to 3D high-resolution LGE-CMR datasets would enable more precise quantification of scar architecture, while incorporating computational electrophysiology may reveal synergistic value between imaging and functional simulations. Such efforts will be critical to refining risk prediction frameworks and translating them into clinically deployable, personalized decision-support systems.

## Methods

### Study population

This study used two independent datasets to develop and validate multivariable survival prediction models for major arrhythmic events in patients with ischemic heart disease. Dataset 1 consisted of a prospective observational registry cohort, whereas Dataset 2 was derived from the REVIVED-BCIS2 multicentre randomized controlled trial. The datasets were used in multiple train-test configurations to evaluate model performance and generalizability across distinct clinical populations.

*Dataset 1* was from prospectively recruited patients referred to the Royal Brompton & Harefield NHS Foundation Trust between 2009 and 2016 for LGE-CMR imaging assessment of known or suspected ischemic heart disease. Imaging was performed on 1.5 Tesla scanners (Siemens Sonata/Avanto) using a standardized protocol. Normal myocardium was nulled to enhance infarct visualization. Quantification of scar infarct and PIZ was performed by a Level 3 accredited operator blinded to outcomes.

Inclusion criteria comprised significant epicardial coronary artery disease (≥75% stenosis in major vessels) or prior myocardial infarction confirmed by revascularization history or imaging evidence. Exclusion criteria were: class I indication for a secondary prevention implantable cardioverter-defibrillator (ICD), myocardial infarction ≤40 days before imaging, severe valvular disease, non-ischemic cardiomyopathy, or absence of myocardial scar. After exclusions and accounting for loss to follow-up, 397 patients with complete imaging and outcome data were included^8,27^.

Dataset 1 was conducted in accordance with the principles of the Declaration of Helsinki and received approval from the National Research Ethics Service under the Cardiovascular Magnetic Resonance for the Assessment of Myocardial Fibrosis Using Delayed Contrast Hyperenhancement study (reference 07/H0708/83; approved 27 March 2008) and the Cardiovascular Biomedical Research Unit Biobank (reference 09/H0504/104+5; approved 3 July 2014). Written informed consent was obtained from all participants before inclusion in the study.

*Dataset 2* was derived from the REVIVED-BCIS2 trial, a prospective, multi- center, randomized controlled trial enroling patients with ischemic LV systolic dysfunction (LVEF ≤35%). Patients were randomized 1:1 to percutaneous coronary intervention plus optimal medical therapy or optimal medical therapy alone and followed up for a median of 3.4 years^43^. REVIVED scans were collected from 34 sites across the UK and included 1.5 and 3T scans, multivendor

Eligibility criteria included: extensive coronary artery disease (British Cardiovascular Intervention Society jeopardy score ≥6), viability in at least four dysfunctional myocardial segments. Patients were excluded if they had had an acute myocardial infarction or acute decompensated heart failure within 4 weeks or 72 hours before randomization respectively. Outcomes were adjudicated by a blinded clinical events committee, with the primary trial endpoints being all-cause mortality and heart failure hospitalization. Arrhythmic events and ICD therapies were captured as secondary endpoints^44^. After exclusions and accounting for loss to follow-up, 424 patients with complete imaging and outcome data were included.

The REVIVED-BCIS2 (Dataset 2) study was approved by the UK Health Research Authority (Westminster Research Ethics Committee, ethics approval reference number: 10/H0802/46), and all procedures were conducted in accordance with applicable ethical and regulatory standards. All participants provided written informed consent. The REVIVED-BCIS2 trial was prospectively registered with the ISRCTN Registry (ISRCTN45979711; registered 20 November 2012) and ClinicalTrials.gov (NCT01920048) before commencement of patient recruitment. (more details^45^).

The study size was determined by the number of eligible participants available in the source datasets. Following application of the predefined inclusion and exclusion criteria, 397 patients from Dataset 1 and 424 patients from Dataset 2 were included. No formal sample size calculation was performed because this was a secondary analysis of existing cohorts. Cases with incomplete imaging, clinical, or follow-up data were excluded before model development and validation. The final analytical datasets therefore contained no missing predictor or outcome values, and no imputation methods were applied.

### Clinical endpoint

The primary endpoint of this study was the occurrence of a major arrhythmic event, defined as sudden cardiac death, aborted sudden cardiac death, haemodynamically unstable ventricular tachycardia, or ventricular fibrillation. In *Dataset 1*, major arrhythmic events were determined from hospital records and coroner reports, with adjudication by independent cardiologists blinded to imaging data. In *Dataset 2*, major arrhythmic events were adjudicated by an independent clinical events committee in accordance with prespecified trial definitions.

Patients who did not experience a major arrhythmic event were censored at the date of last follow-up or study completion. Non-arrhythmic causes of death were treated as censoring events, consistent with a cause-specific hazard modelling framework focused on arrhythmic outcomes. Follow-up time was defined from baseline CMR acquisition (*Dataset 1*) or trial randomization (*Dataset 2*) to either the occurrence of an arrhythmic event or censoring.

**Fig. 7:**
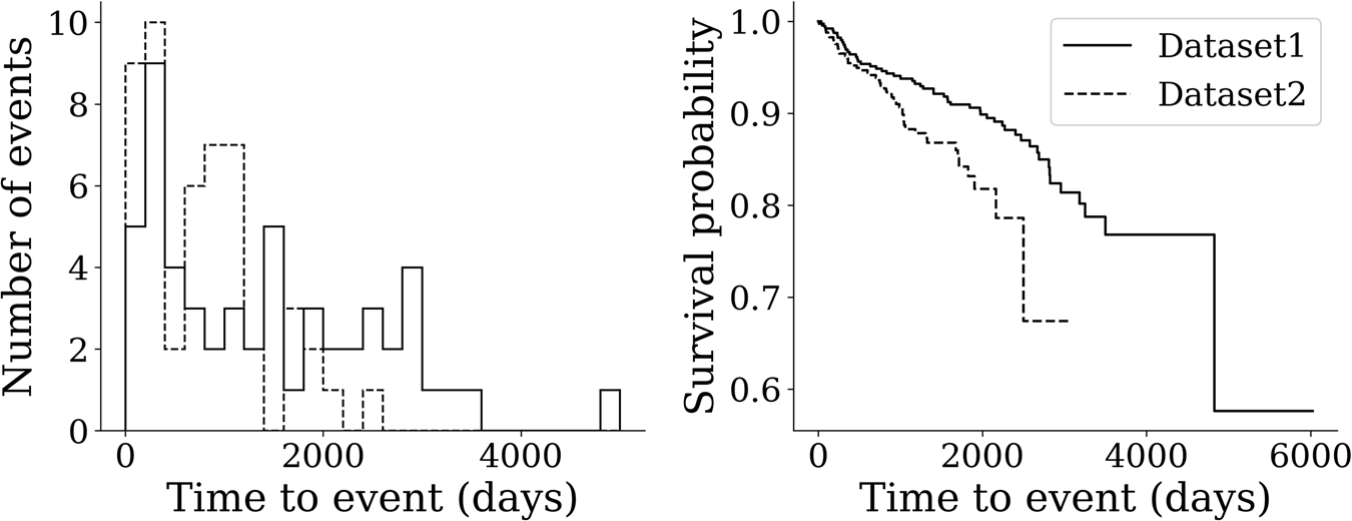
Outcome characteristics of the study populations. (left) Histogram of time to major arrhythmic events in Dataset 1 (solid line) and Dataset 2 (dashed line), shown using 200-day intervals. (right) Kaplan--Meier curves illustrating arrhythmia-free survival in the two datasets. Solid and dashed lines correspond to Dataset 1 and Dataset 2, respectively.

To characterize the temporal distribution of events, we visualized major arrhythmic events across both cohorts. Figure 7 (left) displays the distribution of event times for patients experiencing major arrhythmic events, binned in 200-day intervals. Both datasets demonstrated a concentration of early events, followed by a long right-skewed tail of late occurrences, highlighting heterogeneous risk trajectories. The median follow-up time was 2231 days (IQR: 1707–2812) for Dataset 1 and 1100 days (IQR: 757–1680) for Dataset 2.

In addition, Kaplan-Meier curves were generated to depict arrhythmic-free survival over time (Fig. 7 (right)). These curves illustrate the overall outcome distribution, including censoring, and confirm differences in follow-up duration between the two cohorts. Together, these descriptive analyses highlight the non-uniform timing of arrhythmic events. Survival-based models are appropriate for analysing such time-to-event data, as they naturally account for censored observations and variable follow-up durations.

### Predictors

Quantitative predictors of arrhythmic risk were derived from both imaging and clinical data sources. LGE-CMR imaging was used to characterize myocardial-scar morphology and tissue heterogeneity. For each patient, a stack of short-axis LGE-CMR slices covering the entire LV was processed using semi-automated segmentation based on the full-width at half-maximum (FWHM)^46^ thresholding technique to delineate the infarct scar and PIZ.

**Fig. 8:**
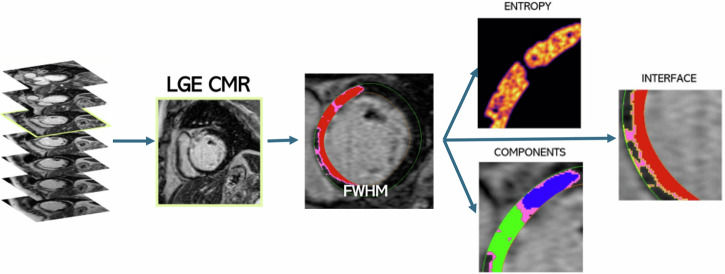
LGE-CMR image processing and feature extraction pipeline. Representative short-axis late gadolinium enhancement cardiovascular magnetic resonance images are segmented using the full-width at half-maximum (FWHM) method to delineate infarct scar and peri-infarct zone (PIZ) regions. Morphological and texture-based features are subsequently extracted for downstream analysis.

From these segmentations, a set of two-dimensional morphological and texture-based features was extracted to capture the structural characteristics of scarred myocardium. Features were computed separately for the infarct scar, the PIZ, and their combined regions (referred to as Total LGE), then aggregated across the LV stack to yield patient-level descriptors. The metrics included entropy (quantifying tissue disorder), number of connected scar components (reflecting scar fragmentation), and myocardial-scar interface area (IA, representing the border length between healthy and fibrotic tissue). Figure 8 illustrates the LGE-CMR processing pipeline, while Supplementary Table 1 defines the selected scar features.

Entropy was computed as a histogram-based measure of intensity distribution within segmented scar regions derived from LGE-CMR, following the methodology described in previous work^27^. This approach quantifies the degree of heterogeneity in signal intensity by capturing the distribution of voxel intensities within the scar and peri-infarct regions.

The extracted features are not standard radiomics texture descriptors, but rather custom, physiologically motivated metrics derived from LGE-CMR segmentation. These include volumetric measures, structural descriptors, and entropy-based features that quantify the spatial organization and heterogeneity of scar and peri-infarct tissue, following previously described methodology. While entropy-based features have shown reproducible behaviour across different contrast agents, it remains uncertain to what extent imaging characteristics such as scanner vendor, magnetic field strength, and LGE acquisition protocols may influence these measurements^27,41^

Table 7 summarizes baseline scar-related features stratified by arrhythmic outcome. Across both datasets, patients who experienced arrhythmic events showed numerically higher component counts, greater entropy values—reflecting increased tissue heterogeneity—and larger myocardial-scar interface areas. Total-LGE entropy and PIZ interface area were elevated in the event groups in both cohorts; however, these differences reached statistical significance only in *Dataset 1*, while *Dataset 2* demonstrated similar directional trends without achieving significance. Taken together, the consistent directionality across datasets is compatible with a potential relationship between scar heterogeneity and arrhythmic risk, although the evidence remains limited in the absence of statistical significance in the second cohort.

**Table 7:** Baseline scar characteristics in patients with and without arrhythmic events across both datasets

Baseline scar characteristics by arrhythmic outcome								
	Dataset 1				Dataset 2			
	Total (*n* = 399)	No Event (*n* = 345)	Event (*n* = 54)	*p* value	Total (*n* = 424)	No Event (*n* = 374)	Event (*n* = 50)	*p* value
Myo. vol (mL)	106 ± 36	102 ± 34	128 ± 38	<0.01	97.6 ± 43.7	97.4 ± 44.4	99.3 ± 38.3	0.85
Scar vol (mL)	15.4 ± 11.3	14.0 ± 10.3	23.0 ± 13.2	<0.01	35.2 ± 21.9	34.7 ± 22.0	39.1 ± 20.6	0.11
PIZ vol (mL)	11.7 ± 7.8	10.9 ± 7.4	15.9 ± 8.6	<0.01	7.92 ± 9.88	7.89 ± 9.99	8.13 ± 9.11	0.87
Total-LGE vol (mL)	27.2 ± 18.2	25.0 ± 16.9	38.9 ± 20.5	<0.01	43.1 ± 24.5	42.6 ± 24.9	47.2 ± 21.1	0.10
Scar comp (%)	42.4 ± 27.5	40.7 ± 26.6	51.8 ± 30.7	<0.01	38.8 ± 41.5	38.8 ± 41.8	38.8 ± 39.3	0.79
PIZ comp (%)	107 ± 67.4	102 ± 65.0	136 ± 73.5	<0.01	110 ± 133	108 ± 131	127 ± 142	0.35
Total-LGE comp (%)	50.6 ± 30.9	49.2 ± 30.4	57.8 ± 32.6	0.02	44.2 ± 52.8	44.0 ± 53.8	45.2 ± 45.5	0.44
Scar entropy	23.8 ± 9.29	23.1 ± 9.24	27.9 ± 8.51	<0.01	36.0 ± 14.5	35.9 ± 15.0	36.5 ± 10.3	0.76
PIZ entropy	18.7 ± 7.23	18.1 ± 7.16	21.8 ± 6.89	<0.01	14.8 ± 14.2	14.7 ± 14.2	15.8 ± 14.1	0.51
Total-LGE entropy	26.6 ± 10.1	25.7 ± 10.0	31.2 ± 9.42	<0.01	37.8 ± 15.3	37.7 ± 15.8	38.3 ± 11.0	0.75
Scar IA	100 ± 63.7	92.6 ± 59.5	108 ± 71.0	<0.01	99.5 ± 86.2	98.2 ± 84.9	109 ± 95.4	0.50
PIZ IA	175 ± 112	165 ± 107	229 ± 127	<0.01	111 ± 142	109 ± 139	125 ± 157	0.61
Total-LGE IA	118 ± 73.1	111 ± 70.6	151 ± 79.6	<0.01	111 ± 103	110 ± 103	117 ± 105	0.59

Continuous variables are expressed as mean ± standard deviation.

Vol. is volume, Comp is components, Ent is entropy, and IA is interface area.

Continuous variables were compared between groups using the two-sided Mann-Whitney *U* test. Categorical variables were compared using the chi-square test, with Fisher’s exact test applied when expected cell counts were less than 5. *P* values reported in Table 7 and Table 8 reflect these comparisons.

**Table 8:** Baseline clinical and echocardiographic characteristics of patients with and without arrhythmic events

Baseline patient characteristics: major arrhythmic event								
	Dataset 1				Dataset 2			
	Total Cohort	Non Event	Event	*p* value	Total Cohort	No Event	Event	*p* value
LVEDV (mL)	218 ± 82.4	208 ± 75.9	272 ± 95.9	<0.01	264 ± 72.2	260 ± 69.8	292 ± 83.1	<0.01
LVEDVi (mL/m^2^)	110 ± 40.7	106 ± 38.0	133 ± 47.2	<0.01	132 ± 34.7	131 ± 33.7	144 ± 39.7	0.02
LVESV (mL)	129 ± 80.2	119 ± 74.3	179 ± 92.8	<0.01	197 ± 66.0	194 ± 63.9	224 ± 75.8	<0.01
LVESVi (mL/m^2^)	65.4 ± 40.5	61.2 ± 38.1	88.2 ± 46.0	<0.01	99.6 ± 32.6	98.0 ± 31.7	111 ± 36.4	0.01
LVEF (%)	45.1 ± 15.8	46.6 ± 15.9	37.0 ± 12.7	<0.01	25.9 ± 7.63	26.1 ± 7.71	24.0 ± 6.78	0.03
Age (years)	64.4 ± 9.79	64.7 ± 9.88	63.0 ± 9.23	0.10	69.6 ± 8.93	69.5 ± 9.09	70.1 ± 7.60	0.57
BSA (m^2^)	1.94 ± 0.28	1.93 ± 0.29	2.02 ± 0.20	0.01	2.00 ± 0.25	1.99 ± 0.24	2.04 ± 0.28	0.25
Sex (%, male)	87.2	86.0	93.8	0.131	87.5	86.6	94.0	0.21
Diabetes (%, positive)	28.8	28.7	29.7	0.985	41.3	42.0	36.0	0.51
NYHA class (%, >I)	34.4	35.2	37.7	0.473	20.5	20.3	22.0	0.92
Prior MI (%, positive)	76.5	75.6	81.2	0.416	9.0	9.1	8.0	1.00

Summary of continuous and categorical patient-level variables for both datasets, stratified by event status. Continuous measures (e.g., LVEDV, LVEF) are presented as mean ± standard deviation. Categorical variables (e.g., sex, diabetes) are shown as percentages.

To complement imaging features, we incorporated a set of clinical variables to capture the broader patient phenotype associated with arrhythmic risk. These included continuous measures such as LVEDV, LVESV, LVEF, age, and body surface area (BSA), as well as categorical indicators including sex, diabetes status, NYHA class, and prior myocardial infarction. Table 8 summarizes these characteristics across both datasets, stratified by arrhythmic outcome. A combined visualization of imaging and clinical predictors is shown in Supplementary Fig. 1.

### Feature selection strategy

To identify informative predictor combinations, a brute-force combinatorial feature selection strategy was employed. This approach systematically explored a large number of feature combinations, enabling comprehensive evaluation of potential interactions and complementary effects between variables without imposing prior assumptions regarding feature importance. Feature selection was performed from a pool of 24 available variables, with candidate subsets containing up to 15 predictors considered during the combinatorial search process. The same feature selection framework was applied consistently across all modelling approaches to ensure fair comparison between CoxPH, RSF, and DeepSurv models and performance was assessed on a validation set using the concordance index (C-index). Although computationally intensive, this approach allowed systematic exploration of feature combinations and enabled the identification of subsets that achieved strong predictive performance. This suggests that interactions and potential non-linear contributions between variables may play an important role in survival prediction.

Alternative feature selection approaches, such as penalized regression or stepwise selection, were not investigated in the present study, as the primary objective was to evaluate predictive performance across modelling frameworks rather than benchmark feature selection techniques.

Feature selection was performed within the training data of each cross-validation fold to avoid information leakage. For each fold, the exhaustive combinatorial search was restricted to the training subset, and the optimal feature subset was selected based on predictive performance. Model performance was then evaluated on the held-out test fold.

### Assessment of predictor correlation and collinearity

Prior to modelling, pairwise correlations among all candidate predictors were assessed to identify highly correlated variables (Supplementary Fig. 2). Correlated variables were retained during model development to allow the feature selection procedure to identify the most informative subset for each modelling approach and dataset configuration.

As feature selection was performed based on predictive performance, the optimisation process implicitly penalizes redundant predictors that do not provide additional information. Consequently, highly correlated variables were not selected together within the same optimal feature subsets, indicating that the selection process naturally avoided redundancy.

### Modelling framework

**Fig. 9:**
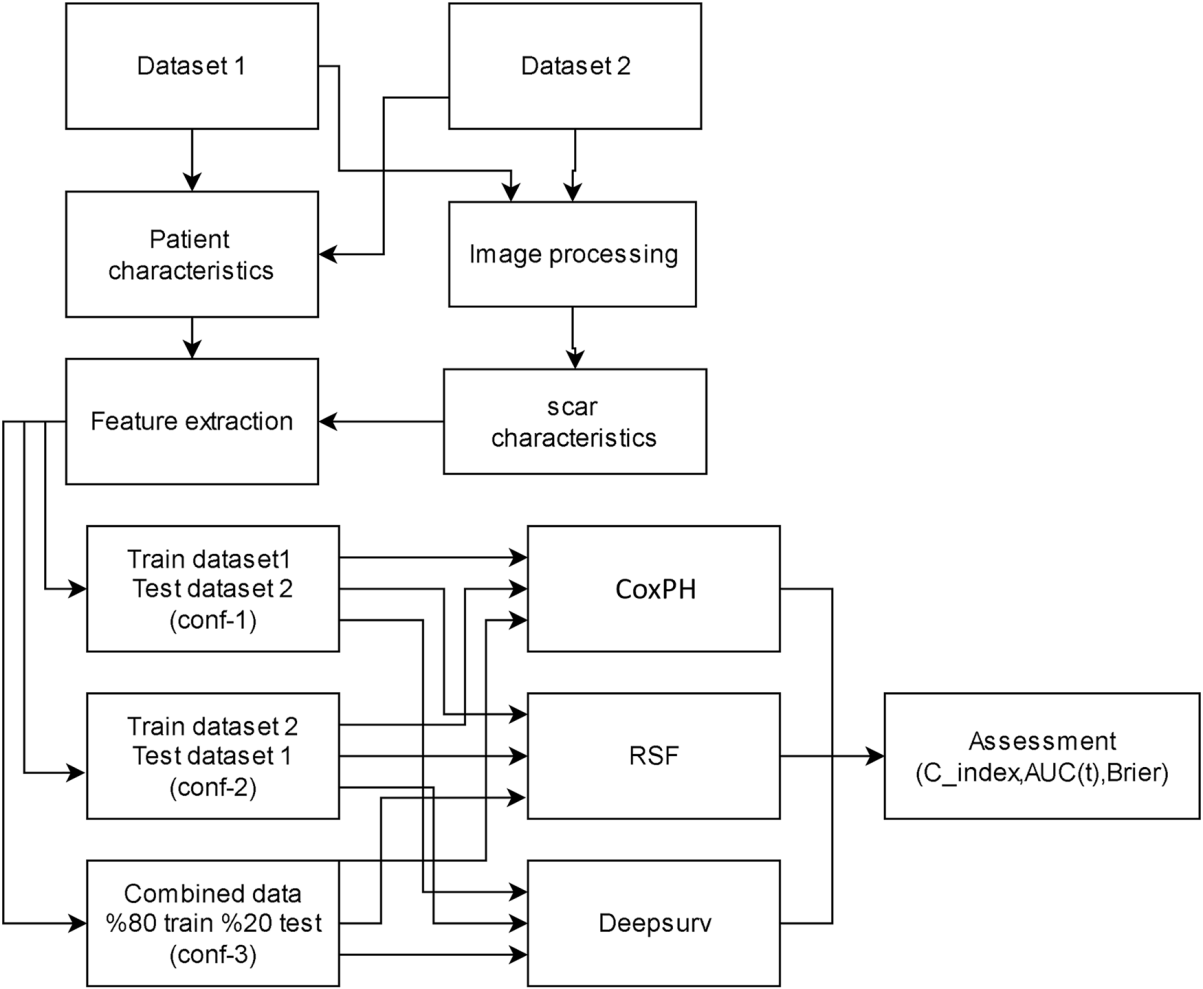
Survival analysis workflow for arrhythmic risk prediction. The methodological pipeline includes data preprocessing, feature extraction, feature selection, model training, and performance evaluation using multiple train--test configurations. Survival prediction was performed using Cox proportional hazards regression (CoxPH), random survival forest (RSF), and DeepSurv models.

Our methodological pipeline integrates multimodal feature extraction with comparative survival modelling across multiple evaluation strategies. The overall workflow is summarized in Fig. 9. Two independent cardiac patient datasets (*Dataset 1* and *Dataset 2*) were processed to derive quantitative imaging biomarkers from LGE-CMR together with clinical covariates. These features were used to train and evaluate three survival analysis models under complementary train-test configurations, enabling assessment of robustness, generalizability, and clinical utility in predicting arrhythmic events.

### Training and evaluation strategies

To rigorously evaluate generalizability, we implemented three complementary train-test strategies:

1. *Train on Dataset 1, test on Dataset 2*: assesses transferability from a real-world observational cohort to a prospective trial population with standardized follow-up (Conf-1).
2. *Train on Dataset 2, test on Dataset 1*: tests whether a model trained on trial data can generalize to a more heterogeneous clinical cohort (Conf-2).
3. *Combined dataset with 80/20 split*: both datasets were merged, and the cohort was randomly partitioned into 80% training and 20% testing. This simulates a real-world scenario where models are trained on diverse populations and evaluated on held-out patients from the same distribution (Conf-3).

In all cases, training and test sets were mutually exclusive. Preprocessing steps (including image segmentation, feature extraction, and normalization) were performed independently within the training folds to avoid data leakage.

### Survival models

We compared three complementary approaches: a classical regression model, a tree-based ensemble, and a neural network extending the CoxPH framework.

The CoxPH^47^ model estimates the hazard function for an individual *i* with covariate vector *x*_*i*_ as:

1 $$h(t| {x}_{i})={h}_{0}(t)\exp \left({\beta }^{\top }{x}_{i}\right),$$

where *h*_0_(*t*) is the baseline hazard and *β* are log-hazard coefficients. Parameters were estimated by maximizing the partial likelihood with optional penalization to improve stability and prevent overfitting. In the penalized setting, the objective function was extended with an elastic-net penalty:

2 $${\ell }_{{\rm{pen}}}(\beta )=\ell (\beta )-\lambda \left[\alpha \parallel \beta {\parallel }_{1}+\frac{1}{2}(1-\alpha )\parallel \beta {\parallel }_{2}^{2}\right],$$

where *λ*≥0 controls the penalty strength and *α* ∈ [0, 1] determines the mixing between L1 (lasso) and L2 (ridge) regularization. Hyperparameters (see Supplementary Table 2) were tuned using cross-validation on the training cohort to maximize Harrell’s concordance index. The model was implemented using the lifelines^48^ Python library.

RSF^23^ is an ensemble of survival trees, each fit on bootstrap samples of the training data using a survival-sensitive splitting rule (log-rank test). For an individual *i*, the ensemble survival estimate is the average across *B* trees:

3 $${\hat{S}}_{{\rm{RSF}}}(t| {x}_{i})=\frac{1}{B}\mathop{\sum }\limits_{b=1}^{B}{\hat{S}}_{b}(t| {x}_{i}).$$

Terminal-node survival estimates were derived using Nelson-Aalen cumulative hazards. RSF was implemented using sksurv^49^ with out-of-bag estimation enabled. Hyperparameters (see Supplementary Table 3) were tuned via internal cross-validation. A fixed random seed ensured reproducibility.

DeepSurv^24^ extends the CoxPH model by replacing the linear risk score *β*^⊤^*x* with a nonlinear function *f*_*θ*_(*x*) parameterized by a neural network:

4 $$h(t| x)={h}_{0}(t)\exp \left({f}_{\theta }(x)\right),$$

where *h*_0_(*t*) is the baseline hazard and *f*_*θ*_(*x*) is learned from data. The network parameters are estimated by minimizing the negative Cox partial log-likelihood:

5 $${\mathcal{L}}(\theta )=-\sum _{i:{\delta }_{i}=1}\left[{f}_{\theta }({x}_{i})-\log \sum _{j\in R({t}_{i})}\exp \left({f}_{\theta }({x}_{j})\right)\right],$$

where *δ*_*i*_ is the event indicator (1 = event, 0 = censored), and *R*(*t*_*i*_) denotes the risk set at time *t*_*i*_.

In our implementation, the network consisted of four hidden layers, each followed by dropout regularization. Hyperparameters (see Supplementary Table 4) were optimized via grid search across hidden layer widths (4–16 units), activation functions (relu, selu, elu, leaky_relu), and dropout rates (0.0–0.3). The output layer contained a single linear unit representing the predicted log-risk. Weights were initialized using the He normal initializer, and the AdamW optimizer was employed for training. Early stopping with a patience of 50 epochs was used to prevent overfitting.

Input features were standardized using StandardScaler. To ensure consistent input size across patients, variable-length feature vectors were zero-padded to a fixed maximum dimension. Missing values in covariates, event time, or event indicator were excluded (complete-case analysis). Model development was performed in TensorFlow/Keras^50,51^ with deterministic operations and fixed random seeds for reproducibility.

Hyperparameter optimisation was performed separately for each experimental configuration. In all cases, tuning was restricted to the training data to avoid information leakage, and model performance was evaluated on the corresponding held-out test set. For Configuration 1 and Configuration 2, hyperparameters were optimised using the training dataset (Dataset 1 or Dataset 2, respectively), and the resulting models were directly evaluated on the independent external test dataset. For Configuration 3, hyperparameters were optimised within the 80% training subset, and the final model was evaluated on the remaining 20% test subset.

### Evaluation

Model performance was evaluated using complementary metrics of discrimination and calibration, capturing different aspects of predictive accuracy over time.

The primary measure of discrimination was the C-index, which quantifies the probability that, in a randomly selected patient pair, the one with the higher predicted risk experiences the event earlier. A higher C-index indicates better ranking ability of the survival model^52^.

Calibration was assessed using the IBS, which summarizes the mean squared differences between predicted survival probabilities and observed outcomes across the entire follow-up period. Lower IBS values correspond to superior calibration and overall predictive accuracy^53^.

To examine temporal discrimination, we computed AUC(*t*) at clinically relevant horizons (2, 5, and 8 years). AUC(*t*) evaluates the model’s ability to correctly distinguish between patients who experience the event before a given time and those who do not, thereby informing short-, intermediate-, and long-term risk stratification. In addition, the mean AUC(*t*) across the full follow-up was reported as a global summary of temporal discrimination^54^.

To quantify statistical uncertainty, all performance metrics were accompanied by 95% confidence intervals estimated using bootstrap resampling of the test set^55^.

Calibration was further evaluated using time-specific calibration plots at 2,5 and 8 years, comparing predicted and observed risks estimated via Kaplan-Meier analysis within risk groups. In addition, calibration error was quantified using the integrated calibration index (ICI), defined as the mean absolute difference between predicted and observed risk^56^.

To further assess the clinical utility of the models, patients were stratified into risk groups based on predicted risk scores derived from each model. Specifically, individuals were divided into low-, intermediate-, and high-risk groups using tertiles^57^ of the predicted risk distribution within the test set. Kaplan-Meier survival curves were then constructed for each risk group, and differences between groups were evaluated using the log-rank test. This approach enables assessment of the models’ ability to discriminate not only between extreme-risk groups but also within the intermediate-risk population, which represents a clinically important subgroup for decision-making.

All metrics were estimated using survival-specific extensions of standard evaluation methods, ensuring proper handling of right-censored data. Results are reported separately for each dataset and training-testing configuration to enable fair comparison between linear and non-linear modelling approaches.

## Supplementary information

Supplementary Information

## Data Availability

Datasets generated and/or analysed during the current study are not publicly available due to patient confidentiality requirements and restrictions imposed by the relevant ethics approvals, but are available from the corresponding author on reasonable request. Dataset 1 was obtained under institutional approvals from the Royal Brompton and Harefield NHS Foundation Trust and is not publicly available. Access requests may be directed to the corresponding author and will be considered subject to institutional and ethical approval. Data from the REVIVED-BCIS2 trial are subject to the trial's data-sharing policies and may be available from the trial investigators upon reasonable request and approval. Custom Python scripts used for data preprocessing, feature extraction, model training, validation, and statistical analysis were developed for this study. The analyses were performed using Python (version 3.10) with standard scientific computing and survival analysis libraries, including NumPy, Pandas, scikit-learn, TensorFlow/Keras, and lifelines. The code required to reproduce the analyses reported in this study is available from the corresponding author upon reasonable request. Access may be subject to institutional approval because portions of the code interface directly with restricted patient datasets that cannot be publicly distributed.

## Code availability

Custom Python scripts used for data preprocessing, feature extraction, model training, validation, and statistical analysis were developed for this study. The analyses were performed using Python (version 3.10) with standard scientific computing and survival analysis libraries, including NumPy, Pandas, scikit-learn, TensorFlow/Keras, and lifelines. The code required to reproduce the analyses reported in this study is available from the corresponding author upon reasonable request. Access may be subject to institutional approval because portions of the code interface directly with restricted patient datasets that cannot be publicly distributed.
