# Supplementary material for: Improving arrhythmic risk prediction using cardiac magnetic resonance within deep learning in ischemic heart disease
Source: NPJ Cardiovasc Health. 2026 Jul 5;3:42. doi: 10.1038/s44325-026-00142-5 (PMC13392232; doi:10.1038/s44325-026-00142-5)
Supplement: Supplementary file 1 — Supplementary Information [file 44325_2026_142_MOESM1_ESM.pdf]

Improving Arrhythmic Risk Prediction using Cardiac Magnetic Resonance Within Deep Learning in Ischemic Heart Disease

Ahmet SEN[1*], Richard E. JONES[2,3], Holly MORGAN[4], Hassan ZAIDI[1], Brian P. HALLIDAY[2,5], Daniel J. HAMMERSLEY[2,5,6], Amedeo CHIRIBIRI[1], Divaka PERERA[3], Sanjay K. PRASAD[2,3], Martin BISHOP[1]

[1]King's College London, School of Biomedical Engineering & Imaging Sciences, London, United Kingdom.
[2]Imperial College London, National Heart and Lung Institute, London, United Kingdom.
[3]Cardiovascular Magnetic Resonance Unit, Royal Brompton and Harefield Hospitals, Guy's and St Thomas' NHS Foundation Trust, London, United Kingdom.
[4]King's College London, British Heart Foundation Centre of Research Excellence at the School of Cardiovascular and Metabolic Medicine & Sciences, London, United Kingdom.
[5]Part of Guy's and St Thomas' NHS Foundation Trust, Royal Brompton and Harefield Hospitals, London, United Kingdom.
[6]NHS Foundation Trust, King's College Hospital, London, United Kingdom.

*Corresponding author(s). E-mail(s): ahmet.1.sen@kcl.ac.uk;

Supplementary Tables

Feature name	Description
Entropy	Degree of disorder within scar tissue, computed using Shannon entropy.
Interface area	Extent of the border between viable myocardium and scar regions, calculated per slice and aggregated across the left ventricle.
Number of components	Number of spatially distinct scar clusters, representing the degree of scar fragmentation.

Supplementary Table 1. Description of selected image-derived scar features extracted from LGE-CMR.

**Hyperparameter**	**Values tested**
Penalty strength (λ)	$10^{-3}, 10^{-2}, 10^{-1}, 1$
Mixing parameter (α)	$0.0, 0.3, 0.5, 0.7, 0.9, 1.0$
Ties handling	`piecewise`, `spline`, `breslow`
Maximum iterations	$10^4, 10^5$

Supplementary Table 2. Hyperparameter grid tested for penalized Cox proportional hazards models.

**Hyperparameter**	**Values tested**
Number of trees	$50, 100, 200$
Minimum samples split	$2, 4, 10$
Minimum samples leaf	$2, 6, 8$
Maximum tree depth	$2, 4, 10$
Random state	$0, 10, 30, 40$
Maximum features	`sqrt`, None

Supplementary Table 3. Hyperparameter grid tested for Random Survival Forest models.

Hyperparameter	Values tested
Number of neurons per hidden layer	$4, 6, 8, 10, 12$
Number of hidden layers	$3, 4, 5, 6$
Activation function	`ReLU`, `ELU`, `LeakyReLU`
Dropout rate	$0.0, 0.1, 0.2$
Batch normalization	on, off
Learning rate	$10^{-4}, 5 \times 10^{-4}, 10^{-3}, 3 \times 10^{-3}$
Optimizer	`Adam`, `AdamW`
Weight decay	$0, 10^{-5}, 10^{-4}, 10^{-3}$
Batch size	$32, 64, 128$

Supplementary Table 4. Hyperparameter grid tested for DeepSurv models.

Supplementary Figures

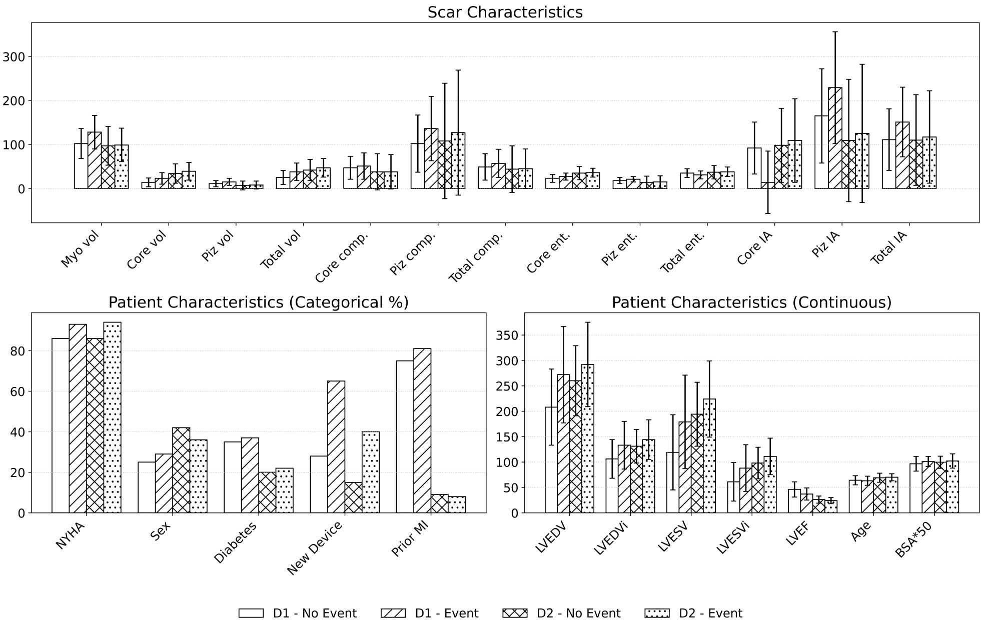

Supplementary Figure 1. Distribution of scar and patient characteristics across cohorts and outcome groups. Top: scar feature distributions for core infarct, peri-infarct zone (PIZ), and combined scar regions. Bottom right: echocardiographic and demographic variables across patients. Bottom left: categorical clinical variables represented as percentages. Colours indicate subgrouping by dataset (D1, Dataset 1; D2, Dataset 2) and event status. Bars represent group means and error bars indicate standard deviations.

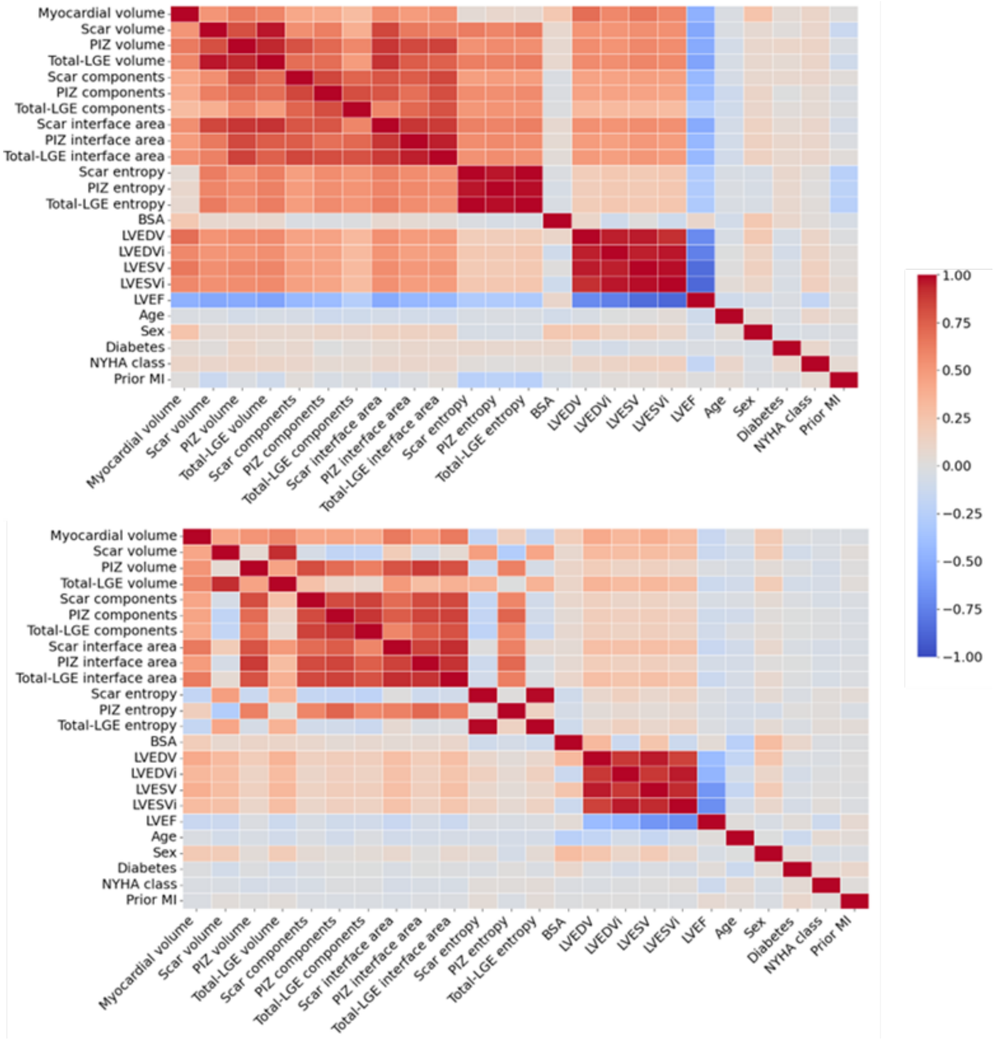

Supplementary Figure 2. Correlation structure of clinical and imaging-derived variables. Correlation matrices are shown for Dataset 1 and Dataset 2 to illustrate relationships among candidate predictors.

TRIPOD Checklist: Prediction Model Development and Validation

Section/Topic	Item		Checklist Item	Page
**Title and abstract**				
Title	1	D;V	Identify the study as developing and/or validating a multivariable prediction model, the target population, and the outcome to be predicted.	1
Abstract	2	D;V	Provide a summary of objectives, study design, setting, participants, sample size, predictors, outcome, statistical analysis, results, and conclusions.	1
**Introduction**				
Background and objectives	3a	D;V	Explain the medical context (including whether diagnostic or prognostic) and rationale for developing or validating the multivariable prediction model, including references to existing models.	2
	3b	D;V	Specify the objectives, including whether the study describes the development or validation of the model or both.	3
**Methods**				
Source of data	4a	D;V	Describe the study design or source of data (e.g., randomized trial, cohort, or registry data), separately for the development and validation data sets, if applicable.	17
	4b	D;V	Specify the key study dates, including start of accrual; end of accrual; and, if applicable, end of follow-up.	18-19
Participants	5a	D;V	Specify key elements of the study setting (e.g., primary care, secondary care, general population) including number and location of centres.	17
	5b	D;V	Describe eligibility criteria for participants.	17
	5c	D;V	Give details of treatments received, if relevant.	N/A
Outcome	6a	D;V	Clearly define the outcome that is predicted by the prediction model, including how and when assessed.	18
	6b	D;V	Report any actions to blind assessment of the outcome to be predicted.	17
Predictors	7a	D;V	Clearly define all predictors used in developing or validating the multivariable prediction model, including how and when they were measured.	19
	7b	D;V	Report any actions to blind assessment of predictors for the outcome and other predictors.	N/A
Sample size	8	D;V	Explain how the study size was arrived at.	18
Missing data	9	D;V	Describe how missing data were handled (e.g., complete-case analysis, single imputation, multiple imputation) with details of any imputation method.	18
Statistical analysis methods	10a	D	Describe how predictors were handled in the analyses.	24
	10b	D	Specify type of model, all model-building procedures (including any predictor selection), and method for internal validation.	24
	10c	V	For validation, describe how the predictions were calculated.	31, 33
	10d	D;V	Specify all measures used to assess model performance and, if relevant, to compare multiple models.	33
	10e	V	Describe any model updating (e.g., recalibration) arising from the validation, if done.	N/A
Risk groups	11	D;V	Provide details on how risk groups were created, if done.	24
Development vs. validation	12	V	For validation, identify any differences from the development data in setting, eligibility criteria, outcome, and predictors.	31
**Results**				
Participants	13a	D;V	Describe the flow of participants through the study, including the number of participants with and without the outcome and, if applicable, a summary of the follow-up time. A diagram may be helpful.	19
	13b	D;V	Describe the characteristics of the participants (basic demographics, clinical features, available predictors), including the number of participants with missing data for predictors and outcome.	20
	13c	V	For validation, show a comparison with the development data of the distribution of important variables (demographics, predictors and outcome).	19
Model development	14a	D	Specify the number of participants and outcome events in each analysis.	19
	14b	D	If done, report the unadjusted association between each candidate predictor and outcome.	N/A
Model specification	15a	D	Present the full prediction model to allow predictions for individuals (i.e., all regression coefficients, and model intercept or baseline survival at a given time point).	N/A
	15b	D	Explain how to the use the prediction model.	N/A
Model performance	16	D;V	Report performance measures (with CIs) for the prediction model.	9
Model-updating	17	V	If done, report the results from any model updating (i.e., model specification, model performance).	N/A
**Discussion**				
Limitations	18	D;V	Discuss any limitations of the study (such as nonrepresentative sample, few events per predictor, missing data).	16
Interpretation	19a	V	For validation, discuss the results with reference to performance in the development data, and any other validation data.	15
	19b	D;V	Give an overall interpretation of the results, considering objectives, limitations, results from similar studies, and other relevant evidence.	15
Implications	20	D;V	Discuss the potential clinical use of the model and implications for future research.	17
**Other information**				
Supplementary information	21	D;V	Provide information about the availability of supplementary resources, such as study protocol, Web calculator, and data sets.	25
Funding	22	D;V	Give the source of funding and the role of the funders for the present study.	25

*Items relevant only to the development of a prediction model are denoted by D, items relating solely to a validation of a prediction model are denoted by V, and items relating to both are denoted D;V. We recommend using the TRIPOD Checklist in conjunction with the TRIPOD Explanation and Elaboration document.
